# Effects of A2E-Induced Oxidative Stress on Retinal Epithelial Cells: New Insights on Differential Gene Response and Retinal Dystrophies

**DOI:** 10.3390/antiox9040307

**Published:** 2020-04-10

**Authors:** Luigi Donato, Rosalia D’Angelo, Simona Alibrandi, Carmela Rinaldi, Antonina Sidoti, Concetta Scimone

**Affiliations:** 1 Department of Biomedical and Dental Sciences and Morphofunctional Imaging, Division of Medical Biotechnologies and Preventive Medicine, University of Messina, 98125 Messina, Italy; rdangelo@unime.it (R.D.); simona.alibrandi@live.it (S.A.); crinaldi@unime.it (C.R.); cscimone@unime.it (C.S.); 2 Department of Biomolecular Strategies, Genetics and Avant-Garde Therapies, I.E.ME.S.T., 90139 Palermo, Italy; 3 Department of Chemical, Biological, Pharmaceutical and Environmental Sciences, University of Messina, 98125 Messina, Italy

**Keywords:** RNA-Seq, RPE, Retinitis pigmentosa, A2E

## Abstract

Oxidative stress represents one of the principal inductors of lifestyle-related and genetic diseases. Among them, inherited retinal dystrophies, such as age-related macular degeneration and retinitis pigmentosa, are well known to be susceptible to oxidative stress. To better understand how high reactive oxygen species levels may be involved in retinal dystrophies onset and progression, we performed a whole RNA-Seq experiment. It consisted of a comparison of transcriptomes’ profiles among human retinal pigment epithelium cells exposed to the oxidant agent N-retinylidene-N-retinylethanolamine (A2E), considering two time points (3h and 6h) after the basal one. The treatment with A2E determined relevant differences in gene expression and splicing events, involving several new pathways probably related to retinal degeneration. We found 10 different clusters of pathways involving differentially expressed and differentially alternative spliced genes and highlighted the sub- pathways which could depict a more detailed scenario determined by the oxidative-stress-induced condition. In particular, regulation and/or alterations of angiogenesis, extracellular matrix integrity, isoprenoid-mediated reactions, physiological or pathological autophagy, cell-death induction and retinal cell rescue represented the most dysregulated pathways. Our results could represent an important step towards discovery of unclear molecular mechanisms linking oxidative stress and etiopathogenesis of retinal dystrophies.

## 1. Introduction

Oxidative stress, recently defined as “a state where oxidative forces exceed the antioxidant systems due to loss of the balance between them”, represents one of the principal inductors of lifestyle-related and genetic diseases [1]. Among them, retinal dystrophies and, in particular, age-related macular degeneration (AMD) and the subgroup of retinitis pigmentosa (RP) are well known to be susceptible to oxidative stress [2,3]. Nowadays AMD is considered the principal cause of visual disability among patients over 50 years [4]. The typical cellular sign of early AMD is represented by drusen, characteristic macular pigmentary deposits, associated with intermediate vision loss [5]. A “dry” and a “wet” form of AMD are currently known, the first more diffused but the second responsible for 90% of acute blindness due to AMD [6]. As previously mentioned, the most relevant risk factor associated with AMD etiopathogenesis is represented by high levels of oxidative stress damaging the macula, generally induced by production of advanced glycation end products (AGE) and exposition to environmental factors [7]. The majority of such effects are also exerted by dysregulation of vascular endothelial growth factor (VEGF), impairment proteins and organelles clearance and glial cell dysfunctions [8]. RP consists of a very heterogeneous group of inherited eye disorders characterized by progressive vision loss [9]. RP has an incidence of 1:4000 people worldwide and represents the most prevalent form of photoreceptor-related diseases [10]. Primary symptoms can already occur in childhood or adolescence and, generally, consist of night blindness and gradual reduction of the visual field due to progressive death of rods. Total blindness, instead, is a feature typical of the late stage of the disease, following degeneration of macula photoreceptors [11]. Rods represent about 95% of all photoreceptors, and oxidative metabolism of fatty acids is their main energy source [12]. Main causes of rod death are genetic mutations, and more than 80 RP-causative genes have been already identified (https://sph.uth.edu/RetNet/sum-dis.htm#B-diseases), even if a relevant number of them are still unknown [13]. Conversely, cone degeneration is usually a late event frequently resulting from cytotoxic effects of high oxygen levels in the retina after rod reduction. Thus, oxidative damage is considered the first cause of cone apoptosis and progressive vision loss [14]. Interestingly, AMD and RP can also arise due to mutations in genes expressed in other retinal cell types, such as *MERTK* [15], *RLBP1* [16] and *RPE65* [17] expressed in retinal pigment epithelium (RPE). Originally, only a trophic function was hypothesized for RPE cells. Nowadays, it is well known that RPE is a monolayer of neural-crista-derived pigmented epithelial cells interacting with Bruch’s membrane and choriocapillaris on the basolateral side and with the outer segments of the photoreceptors on the apical one [17]. RPE plays many vital roles for photoreceptor cells and the most fascinating certainly is the protection from oxidative stress [18]. Recent studies confirmed a high level of reactive oxygen species (ROS) in RPE, and fatty acids are one of their molecular targets. If oxidized, they may impair transduction pathways and gene expression [19]. Although fatty acid oxidation was already confirmed to cause macular degeneration, oxidative stress mechanism in RP development requires further clarifications [20]. Therefore, to better understand how high ROS levels may lead to retinal dystrophies onset and progression, we performed a comparison of transcriptomes’ profiles among human RPE cells exposed to the oxidant agent N-retinylidene-N-retinylethanolamine (A2E). A2E is a toxic bis-retinoid that derives from the condensation and the oxidation of the trans-retinal [21]. Throughout life, A2E and other complex lipids accumulate and form lipofuscin in the RPE, ultimately determining photoreceptor death [22]. Additionally, A2E photo-oxidation is able to generate singlet oxygen, a highly reactive molecule that contributes to the increase of level of toxic metabolites such as epoxides and endoperoxides [23]. Moreover, lipid peroxidation is also responsible for A2E cleavage that releases cytotoxic reactive aldehydes. These reactive species could affect the lipid membranes fluidity and can damage the DNA and the cellular proteins [24]. On these bases, it was hypothesized that lipofuscin, A2E and its oxidized metabolites could accumulate if the cellular antioxidant system is unable to fight the oxidative-damaged lipids in rods and cones. Consequently, accumulation and photo-oxidation of A2E could lead to several retinal dystrophies, like already established for age-related macular degeneration (AMD) [25]. Various studies based on ARPE-19 cell line present pharmacological solution to retinal dystrophies. The same cell line is also used to identify new candidate compounds able to protect RPE against A2E oxidation [26]. As highlighted in the manuscript, the treatment with such a compound determines relevant differences in gene expression and splicing events, involving several new pathways probably related to retinal degeneration.

## 2. Materials and Methods

### 2.1. Cell Culture

Human RPE-derived Cells (H-RPE—Human Retinal Pigment Epithelial Cells, Clonetics™, Lonza, Walkersville, USA) were grown in T-75 flasks containing RtEGM™ Retinal Pigment Epithelial Cell Growth Medium BulletKit^®^ (Clonetics™, Lonza, Walkersville, MD, USA) with 2% *v*/*v* fetal bovine serum (FBS), 1% of penicillin/streptomycin and incubated at 37 °C with 5% CO_2_. H-RPE cells were then plated into 96-well plates (4 × 10^4^ cells/well) and cultured for 24 h to reach confluence before treatment. Subsequently, A2E was added to a final concentration of 20 μM for 6 h before rinsing with medium. Control cell groups were incubated without A2E. Confluent cultures were transferred to PBS supplemented with calcium, magnesium and glucose (PBS–CMG) and then exposed to blue light emitted by a tungsten-halogen source (470 ± 20 nm; 0.4 mW/mm^2^) for 30 min to induce phototoxicity of A2E and incubated at 37 °C. The 1–3 generation of subcultured RPE cells were used in this study. 

### 2.2. MTT Assay

Cell viability was determined by mitochondrial-dependent reduction of methylthiazolyldiphenyl-tetrazolium bromide (MTT) (Sigma-Aldrich, St. Louis, MO, USA) to formazan-insoluble crystals. Briefly, 10 μL of 5 mg/mL of MTT in PBS was added to the cells following the A2E treatment. After incubation at 37 °C for 2 h, 100 μL of 10% SDS in 0.01 mol/L HCl was added to dissolve the crystals and incubated for 16 h. The absorbance was measured in a Dynatech microplate reader at 570 nm. Results were expressed as percentage of viable cells normalized with control conditions in the absence of A2E.

### 2.3. Total RNA Sequencing

Total RNA was extracted by TRIzol^TM^ Reagent (Invitrogen^TM^, ThermoFisher Scientific, Waltham, MA, USA), following manufacturer’s protocol, and quantified at Qubit 2.0 fluorimeter by Qubit^®^ RNA assay kit (Invitrogen^TM^, ThermoFisher Scientific, Waltham, MA, USA). The RNA-seq samples consisted of 3 factor groups, represented by Human RPE cells, before the treatment with A2E and at 2 following different time points of 3 h and 6 h, respectively. For each group, 3 biological replicates were considered, for a total of 9 samples. The selection of 3 h and 6 h time points was based on previous experiments realized by our research group (unpublished data), confirmed by outcomes from MTT assay in this work. Such results highlighted that in wider time intervals, the death rate of oxidative stressed cells might be so high as to invalidate the following expression analysis. Libraries were generated using 1 µg of total RNA by the TruSeq Stranded Total RNA Sample Prep Kit with Ribo-Zero H/M/R (Illumina, San Diego, CA, USA), according to manufacturer’s protocols. Sequencing runs were performed on an HiSeq 2500 Sequencer (Illumina, San Diego, CA, USA), using the HiSeq SBS Kit v4 (Illumina, San Diego, CA, USA). The experiment was repeated thrice.

### 2.4. Quality Validation and Read Mapping

Sequence reads were generated from RPE-specific cDNA libraries on the Illumina HiSeq 2500 Sequencer. Obtained raw sequences were filtered to remove low-quality reads (average per base Phred score <30) and adaptor sequences. The quality of analyzed data was checked using FastQC (v.0.11.9) (https://www.bioinformatics.babraham.ac.uk/projects/fastqc/) and QualiMap (v.2.2.1) [27], while trimming was realized by Trimmomatic (v.0.39). Filtered data were then mapped by CLC Genomics Workbench v.20.0 (https://digitalinsights.qiagen.com/products-overview/analysis-and-visualization/qiagen-clc-genomics-workbench/) against the Homo sapiens genome hg38 and the RNA database v.91, on Ensembl database. RNA-seq analysis was conducted using the following settings: quality trim limit = 0.01, ambiguity trim maximum value = 2. Map to annotated reference was as follows: mismatch cost = 2, insertion and deletion costs = 3, minimum length fraction and minimum similarity fraction = 0.8, maximum number of hits for a read = 10, strand-specific = both, expression value = TPM. Raw data are available upon request.

### 2.5. Gene Expression and Statistical Analysis

Mapped reads were quantified by alignment-dependent and alignment-independent methods. The first approach uses the expectation-maximization (EM) algorithm [28] in order to determine expressions even in cases where the majority of reads map equally well to multiple genes or transcripts. Once the algorithm has converged, every non-uniquely mapping read was assigned randomly to a particular transcript according to the abundances of the transcripts within the same mapping. The transcript per million reads (TPM) values were, then, computed from the counts assigned to each transcript. The second method has a higher accuracy for the point expression estimation and also allows the user to bootstrap the expression quantification to get an estimate of the technical variability. This approach was applied by the Salmon tool [29] using the transcript fasta files downloaded from GENCODE v.32 (gencode.v32.transcripts.fa). Salmon was run with the following settings for the RNA-seq data: quant –index/index --libType U --unmatedReads /single.fastq --incompatPrior ‘0.0’ --biasSpeedSamp ’5 ’ --fldMax ‘1000’ --fldMean ‘250’ --fldSD ‘25’ --forgettingFactor ‘0.65’ --maxReadOcc ‘100’ --numBiasSamples ‘2000000’ --numAuxModelSamples ‘5000000’ --numPreAuxModelSamples ‘5000’ --numGibbsSamples ‘0’ --numBootstraps ‘0’ --thinningFactor ‘16’ --sigDigits ‘3’ --vbPrior ‘1e-05’ -o/output. Once obtained, Salmon outputs were imported using tximport R package version 1.10.0 and lengthScaledTPM method [30] to generate read counts and Transcripts Per Million (TPMs). Low expressed transcripts and genes were filtered based on the data mean–variance trend analysis. The expected decreasing trend between data mean and variance was observed when expressed transcripts were determined as which had ≥1 of the 9 samples with count per million reads (CPM) ≥1, which provided an optimal filter of low expression. A gene was expressed if any of its transcripts with the above criteria were expressed. The trimmed mean of M-values (TMM) method was used to normalize the gene and transcript read counts to $log_{2}$-CPM [31]. The principal component analysis (PCA) plot showed that the RNA-seq data did not have distinct batch effects, so it was possible to proceed with downstream analyses.

### 2.6. DE, DAS and DTU Analysis

Limma R package was used for differential expression analyses [32]. General linear models were established to compare gene and transcript expression changes at the different conditions of experimental design, setting the contrast groups as 0 h.untreated versus 3 h.treated, 0 h.untreated versus 6 h.treated, 3 h.treated versus 6 h.treated, 0 h.untreated versus (3 h.treated + 6 h.treated)/2. For differentially expressed (DE) genes/transcripts, the $log_{2}$ fold change ($L_{2}FC$) of gene/transcript abundance were calculated based on contrast groups and significance of expression changes were determined using the t-test [33]. *P*-values of multiple testing were adjusted with BH to correct false discovery rate (FDR) [34]. A gene/transcript was significantly DE in a contrast group if it had adjusted *p*-value <0.01 and $L_{2}FC$ ≥1. At the alternative splicing level, differential transcript usage (DTU) transcripts were determined by comparing the $L_{2}FC$ of a transcript to the weighted average of $L_{2}FCs$ (weights were based on their standard deviation) of all remaining transcripts in the same gene. A transcript was determined as significant DTU if it had adjusted *p*-value <0.01 and ΔPS ≥0.1. For differentially alternative spliced (DAS) genes, $L_{2}FC$ of each individual transcript was compared to gene level $L_{2}FC$, which was calculated as the weighted average of $L_{2}FCs$ of all transcripts of the gene. Then *p*-values of individual transcript comparisons were summarized to a single gene level *p*-value with F-test. A gene was significantly DAS in a contrast group if it had an adjusted *p*-value <0.01 and any of its transcript had a Δ Percent Spliced (ΔPS) ratio ≥0.1. Finally, time points (0 h, 3 h, 6 h) in groups (untreated, treated) were used for time-series trend analysis. Natural Cubic Spline method with degree of freedom was used to generate time-series trend (Figure S1).

### 2.7. Gene-Enrichment and Functional Pathway Analysis

The up- and down-regulated genes were analyzed by the Database for Annotation, Visualization and Integrated Discovery (DAVID) 6.8 [35]. This tool is based on more than 40 annotation categories, including GO terms to protein–protein interactions, from disease associations to gene functional summaries, and many others. In DAVID annotation system, EASE Score, a modified Fisher Exact P-Value, is adopted to measure the gene-enrichment in annotation terms. The EASE score provides a conservative adjustment to the Fisher exact probability that weights significance in favor of themes supported by more genes. The EASE score is calculated by penalizing (removing) one gene within the given category from the list and calculating the resulting Fisher exact probability for that category.

### 2.8. Selection of Single-Pathway “Master genes” and Selection of Retinitis Pigmentosa Candidate Genes by ToppGene Prioritization

In order to highlight new candidate genes involved into retinitis pigmentosa, based on oxidative-related candidate pathways, we chose the 15 most altered genes for each one. Firstly, we considered them for each time point; then, we chose the commons in all time points. Subsequently, chosen genes underwent prioritization by ToppGene (https://toppgene.cchmc.org), a web-based tool able to classify a selected group of candidate genes from a large set of genes correlated with a pathology, giving each one a score. The score is based on the intersection of data from various databases of annotations related to cellular and physiological functions, analyzing complex networks shared between genes already known to cause the disease (training genes) and candidate genes (test genes). Training genes were obtained from RetNet online database.

### 2.9. Data Validation by qRT-PCR.

Ten most dysregulated mRNAs from candidate genes previously identified were selected and validated by quantitative Real-Time-Polymerase Chain Reaction (qRT-PCR), in order to validate RNA-Seq data. Reverse transcription was carried out according to the manufacturer’s protocol of GoScript™ Reverse Transcription System (Promega, Madison, WI, USA). The obtained cDNA was subjected to RT-PCR in the ABI 7500 fast sequence detection system (Applied Biosystems, Foster City, CA, USA), using BRYT-Green-based PCR reaction. PCR amplification was performed in a total reaction mixture of 20 μL containing 20 ng cDNA, 10 μL 2 × GoTaq1qPCR Master Mix (Promega, Madison, WI, USA) and 0.2 μM of each primer. PCR was run with the standard thermal cycle conditions using the two-step qRT-PCR method: an initial denaturation at 95 °C for 30 s, followed by 40 cycles of 30 s at 95 °C and 30 s at 60 °C. Each reaction was replicated six times, considering all analyzed time points (18 samples), and the average threshold cycle (Ct) was calculated for each replicate. The expression of mRNAs was calculated relative to expression level of endogenous control β-actin, and the relative expression of genes was calculated using the 2^−ΔΔCt^ method [36]. The results were shown as the mean ± SEM (Standard Error of Mean). Statistical significance was determined by analysis of variance between groups (ANOVA), followed by Bonferroni post-hoc test. Finally, a linear regression analysis was performed to check the correlation of the FC of the gene expression ratios between qRT-PCR and RNA-Seq. The statistical analyses were all performed using IBM SPSS 26.0 software (https://www.ibm.com/analytics/us/en/technology/spss/). Adjusted *p*-values <0.05 were considered statistically significant. The research was approved by the Scientific Ethics Committee of the Azienda Ospedaliera Universitaria—Policlinico “G. Martino” Messina.

## 3. Results

### 3.1. MTT Cell Viability Assay Results

**Figure 1:**
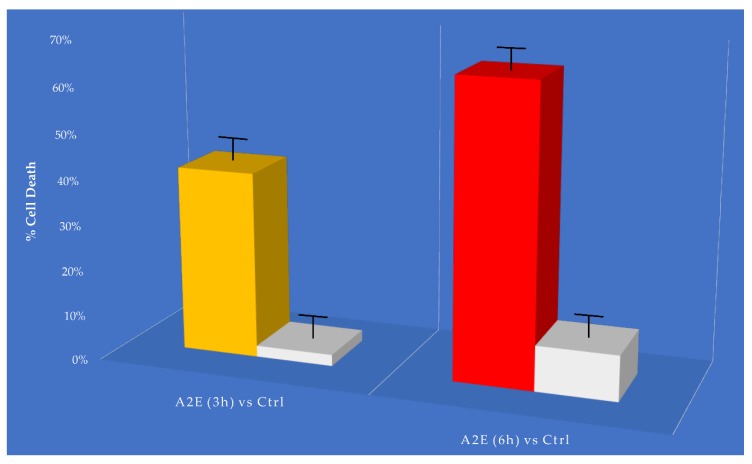
MTT determination of A2E treatment in retinal pigment epithelium (RPE) cells. Cell death was assessed at considered time points (3 h and 6 h) in A2E treated samples compared to basal untreated group. Results are shown as mean ± standard error of mean (*n* = 3). *p*-value < 0.05.

The MTT cell viability assay showed a relevant and different trend in RPE-treated cells versus control. The addition of A2E to cultures led to a dose-dependent increase in cell death percentage (Figure 1).

### 3.2. Sequencing Analysis and Mapping Statistics

RNA sequencing carried out on Illumina HiSeq 2500 yielded a total of 96,346,180 quality reads (mean mapping quality = 29) with a percentage of 67.8% uniquely mapped. A total of 16,173 genes and 69,653 transcripts were identified out of 60,609 and 227,462 reference counterparts, respectively, considering the whole human transcriptome. The annotated reference assembly v.32 (GRCh38.p13) was downloaded from GeneCode FTP server (ftp://ftp.ebi.ac.uk/pub/databases/gencode/Gencode_human/). All previous mapping statistics were based on average values calculated for all three replicates in each time point. Details are available in Figure S2.

### 3.3. Analysis of Gene Expression Profile of RPE Cells

**Figure 2:**
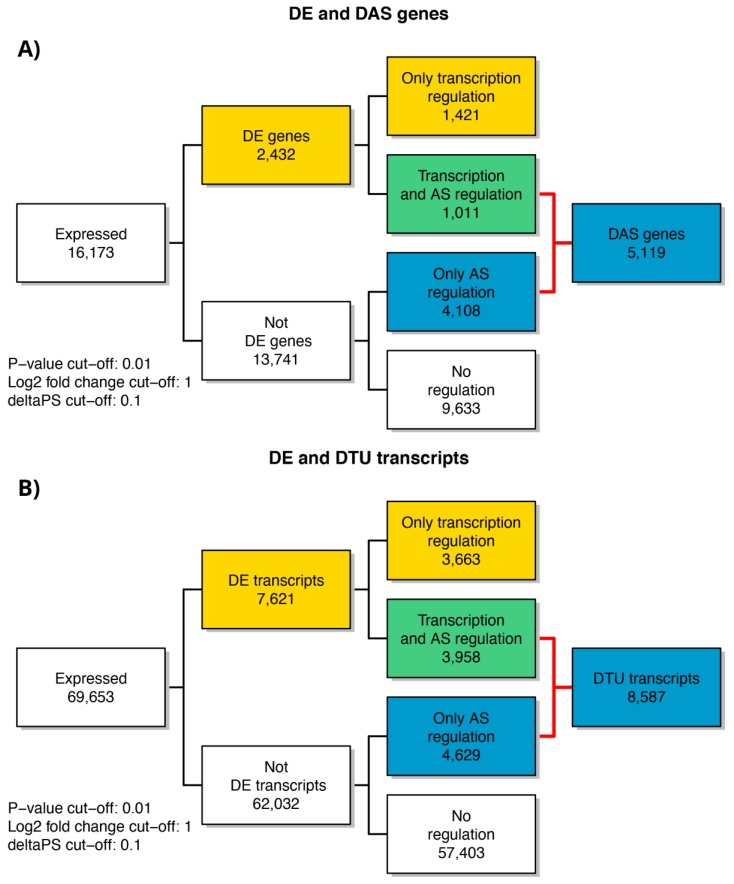
Summary figure of expressed genes and significant DE, DE + DAS, DE + DTU, DAS and DTU genes from analysis of the considered time points of RPE cell data. (**A**) Number of genes regulated only by transcription (DE), only by alternative splicing (DAS) and by both transcription and alternative splicing (DE + DAS); (**B**) number of transcripts regulated only by transcription (DE), only by alternative splicing (DAS) and by both transcription and alternative splicing (DE + DAS). DTU = Differential Transcript Usage. AS = Alternative Splicing.

**Figure 3:**
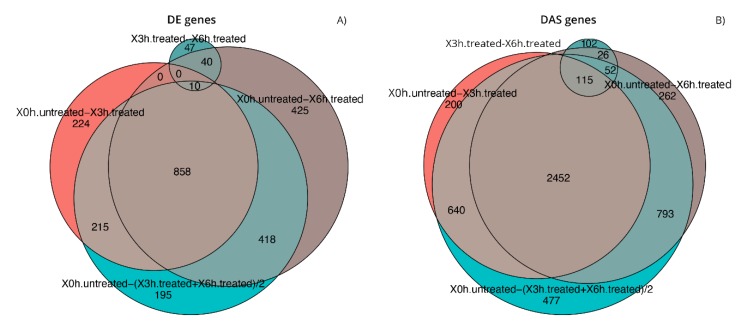
Comparison of the gene lists generated during differential expression analyses. Euler diagrams of DE (**A**) and DAS (**B**) genes identified during expression analyses in considered conditions of experimental design, setting the contrast groups as 0 h.untreated versus 3 h.treated, 0 h.untreated versus 6 h.treated, 3 h.treated versus 6 h.treated, 0 h.untreated versus (3 h.treated + 6 h.treated)/2.

As previously cited, in our transcriptome study, 16,173 genes (Table S1) and 69,653 transcripts (Table S2) were expressed with log_2_-CPM values ≥1 considered as the average value per sample. We first analyzed differential expression at the gene level (DE) (Figure 2a and Table S3). Considering the stringent criteria we have chosen, we identified 2432 genes that were significantly differentially expressed in response to A2E treatment. Of these, 59.7% resulted as up-regulated, while 40.3% down-regulated, showing a decreasing time-dependent trend for up-regulated ones and an increasing trend for down-regulated ones. Afterward, the analysis of transcript-level data allowed us to identify genes that were DAS between the contrast groups (Figure 2b and Table S4). We detected 5119 DAS genes, of which 1101 were also DE genes (regulated by both transcription and AS) and 4108 were regulated by AS only. Therefore, considering all 6540 genes that showed significantly altered levels of differential gene and/or transcript-level expression, 78.3% were differentially alternatively spliced, highlighting a consistent response to the induced oxidative stress. Furthermore, to identify the specific transcripts that characterize a gene as DAS, a DTU analysis was performed (Figure 2b and Table S5). Globally, around 12% (8587) of expressed transcripts were classified as DTU. The next step consisted in the analysis of early response to the induced oxidative stress in comparison with the response to late treatment with A2E. About 40% (858) and 55% (2457) of DE and DAS genes, respectively, resulted as common to both time point observations, while the residual was unique to 3 h and 6 h (Figure 3). Consequently, it is evident how changes in gene-level expression and alternative splicing occurred throughout the whole period, either transiently (occurring after 3 h and returning to initial level after 6 h), occurring later (only after 6 h from treatment) or enduring throughout the whole period. Such results probably suggest the different responses of RPE cells as a growing resistance to oxidative stress stimuli in a time-dependent manner.

### 3.4. DE and DAS Genes Highlighted Different Functionality Patterns

**Figure 4:**
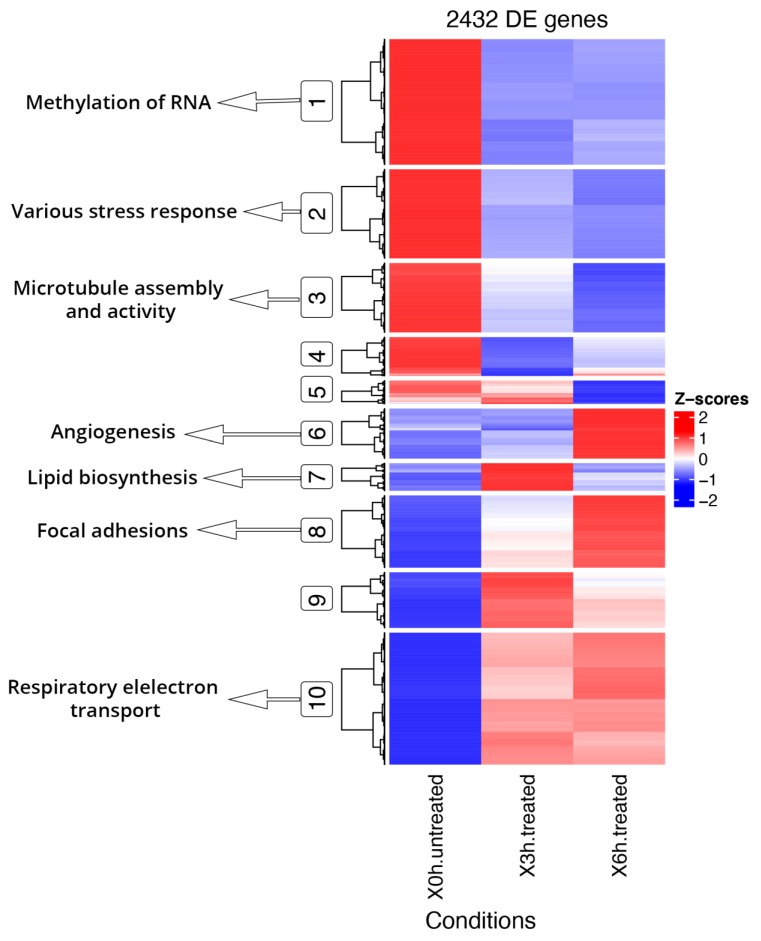
Hierarchical clustering and heatmap of DE genes and Key GO terms. DE genes show segregation into 10 coexpressed clusters, of which the main ones related to oxidative stress were highlighted (circled) and linked to GO specific terms. Full results of GO enrichment analyses are shown in Tables S6 and S7. The z-score scale represents mean-subtracted regularized log-transformed transcripts per million (TPMs).

**Figure 5:**
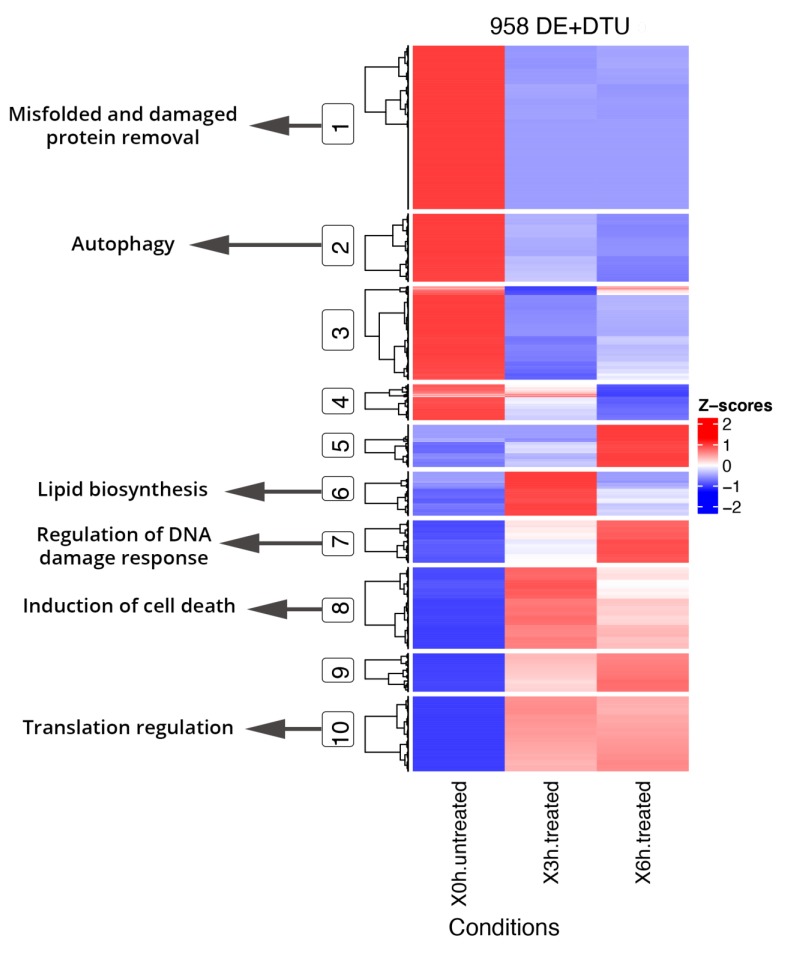
Hierarchical clustering and heatmap of DE genes + DTU transcripts from DAS genes and Key GO terms. DE genes and DTU transcripts from DAS genes show segregation into 10 coexpressed clusters, of which the main ones related to oxidative stress were highlighted (circled) and linked to GO specific terms. Full results of GO enrichment analyses are shown in Tables S6 and S7. The z-score scale represents mean-subtracted regularized log-transformed TPMs.

Functional enrichment analyses of DE and DAS genes showed relevant differences, as already supposed by the overlap of only 1011 genes (Figure 2). The most significantly enriched terms for DE genes were linked to vascular events and very heterogeneous responses to oxidative stress. As evidenced by hierarchical clustering of total gene expression levels of DE genes, adaptive, transient and late expression profiles followed the A2E induced stress, and an analog response was highlighted by transcript expression profiles of individual DE genes (Figure 4). Functional annotation of individual DE genes clusters was associated with methylation of RNA (cluster 1), various stress response (cluster 2), microtubule assembly and activity (cluster 3), angiogenesis (cluster 6), lipid biosynthesis (cluster 7), focal adhesions (cluster 8) and respiratory electron transport (cluster 10). For DAS genes, the most enriched functional terms deal with alteration of cellular proliferation and cell death. Hierarchical clustering of the DTU transcripts and expression profiles of individual DAS genes also evidenced heterogeneous response profiles. Functional annotation of DTU clusters of genes revealed enrichment of terms related to misfolded and damaged protein removal (clusters 1 and 3), autophagy (cluster 2), lipid biosynthesis (cluster 6), regulation of DNA damage response (cluster 7), induction of cell death (cluster 8) and translation regulation (cluster 10) (Figure 5).

### 3.5. Early Cellular Response to Induced Stress Mainly Involves Pre-mRNA Splicing and Glycolysis-Related DE and DAS Genes

**Figure 6:**
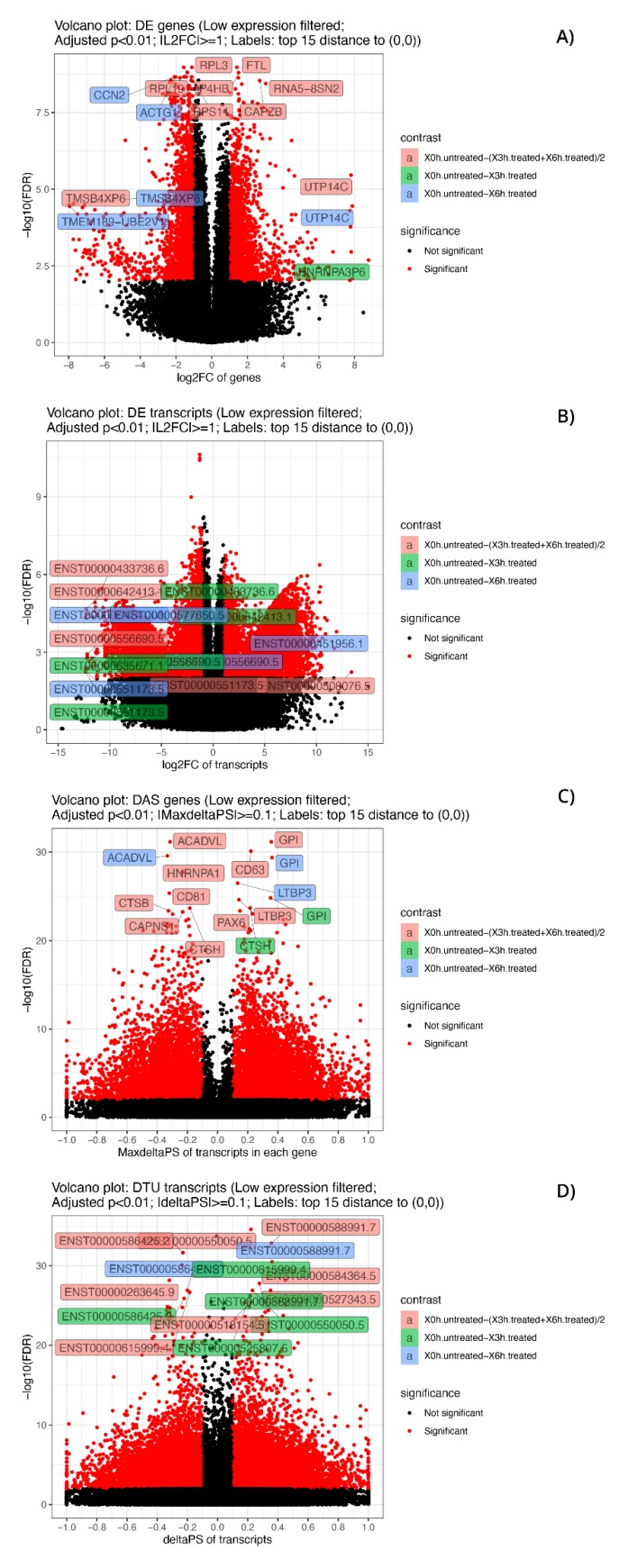
Volcano plots of significant DE and DAS genes and of DE and DTU transcripts. Volcano plots of significant (adjusted *p*-value < 0.01) DE genes (**A**), DE transcripts (**B**), DAS genes (**C**) and DTU transcripts (**D**). The low expressed genes and transcripts were filtered. The top 15 considered elements with the smallest corrected *p*-values and bigger fold-changes are highlighted, and different colors refer to different contrast groups. DE genes: log2FC vs. −log10(FDR) at gene level; DAS genes: maximum ΔPS of transcript in a gene vs. −log10(FDR) at gene level; DE transcripts: log2FC vs. −log10(FDR) at gene level at transcript level and DTU transcripts: ΔPS vs. −log10(FDR) at transcript level.

The applied statistical model used in our analyses permitted us to determine precisely at which time point each DE and DAS gene first showed a significant change, together with the magnitude and course of that change. The top 15 significant genes or transcripts belonging to the considered groups were deeply investigated, allowing the identification of new candidate genes and specific pathways possibly involved in retinal dystrophy etiopathogenesis (Figure 6). After the first 3 h of treatment, only one gene (*HNRNPA3P6*) reached a relevant DE status, and it is involved in cytoplasmic trafficking of RNA and pre-mRNA splicing. Main DAS genes that showed early important differences (*CTSH* and *GPI*), instead, were related to glycolysis, and the same biochemical pathway was the most correlated to initial DTU transcripts expression dysregulation (ENST00000615999.4, ENST00000588991.7, ENST00000586425.2, ENST00000525807.5 and ENST00000550050.5).

### 3.6. Late RPE Cell Response to A2E Treatment Could Impair Bioenergetic Specific Reactions, Extracellular Matrix Integrity and Neurotransmission-Related DE and DAS Genes

**Figure 7:**
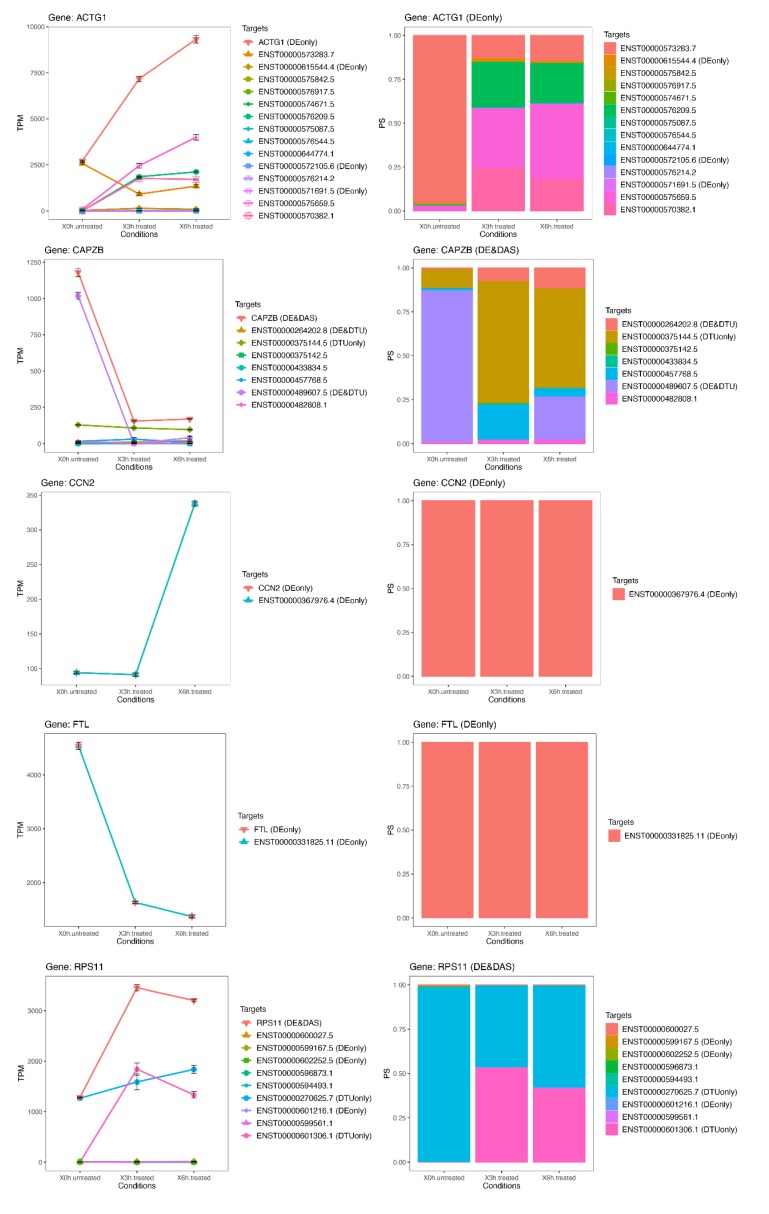
Expression profiles of most important DE genes/transcripts; first cluster associated with newly discovered pathways linked to oxidative stress. Detailed gene/transcript expression profiles across the time course of the first cluster of most important DE genes (ACTG1, CAPZB, CCN2, FTL and RPS11) linked to newly identified candidate pathways linked to oxidative stress.

**Figure 8:**
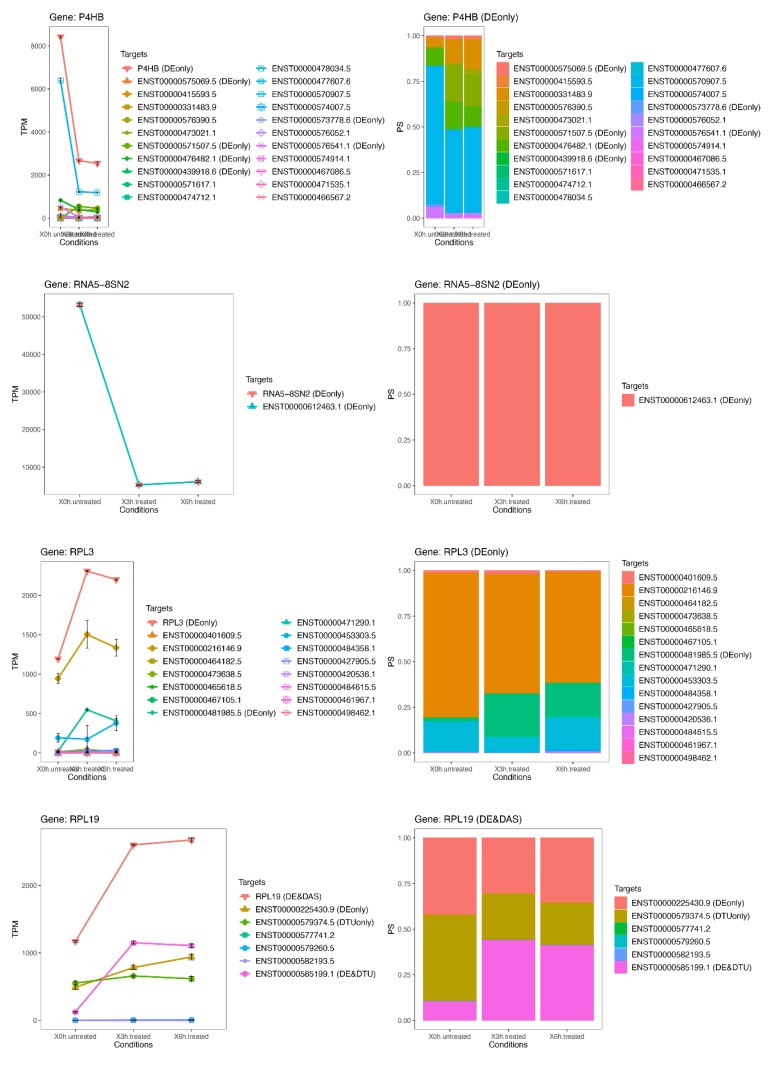
Expression profiles of most important DE genes/transcripts secondo cluster associated with newly discovered pathways linked to oxidative stress. Detailed gene/transcript expression profiles across the time course of the second cluster of most important DE genes (*P4HB*, *RNA5-8SN2, RPL3* and *RPL19)* linked to newly identified candidate pathways linked to oxidative stress.

While the number of significant and relevant DE genes was limited in the early stage of treatment, it grew over time, reaching a climax at 6 h (Figure 6). At this time point, many of the DE genes (*ACTG1*, *CCN2*, *RPL19*, *RPL3*, *P4HB*, *RPS11*, *FTL*, *CAPZB* and *RNA5-8SN2*) (Figure 7 and Figure 8) resulted as linked to new particular pathways, as iron metabolism, plasma lipoproteins assembly and F-actin capping. Furthermore, several over-expressed DE genes at 6 h (*TTC8*, *ARL3*, *REEP6*, *GUCA1B* and *PDE6G*) resulted as associated with alternative splicing of retina-preferred gene transcripts. About DAS genes, instead, the most significant ones (*ACADVL*, *GPI* and *LTBP3*) resulted as correlated to already-involved pathways (e.g., glycolysis). However, more interestingly, the same genes were together with DTU transcripts (ENST00000586425.2 and ENST00000588991.7), but even more interestingly, with other biochemical activities regarding oxidative processes in mitochondria (e.g., fatty acids reactions) and low conductance of potassium channels (as evidenced by DE transcripts ENST00000577650.5, ENST00000451956.1, ENST00000556690.5 and ENST00000551173.5).

### 3.7. The Transcriptome Comparison between Untreated (Time Zero) and Treated (3 h + 6 h) Rpe Cells Revealed the Possible Impairment of Retinal Cells Crosstalk and Synapses, Leading to Rescue or Cell Death

Many DE and DAS genes only showed significant differences only if analyzed during the whole considered treatment period. Among these DE genes, five (*CCN2*, *ACTG1*, *UTP14C*, *TMSB4XP6* and *TMEM189-UBE2V1*) were linked to extracellular matrix constituent secretion and cellular junctions, as well as to misfolded protein ubiquitination. Furthermore, the number of DAS genes and DTU transcripts changed during treatment was very high. The first ones (*ACADVL*, *GPI*, *HNRNPA1*, *CD81*, *CD63*, *CTSB*, *CTSH*, *LTBP3*, *CAPNS1* and *PAX6*) were enriched in terms related to neuropeptide catabolic processes and extracellular vesicles in the crosstalk of cells, while the second ones (ENST00000586425.2, ENST00000550050.5, ENST00000588991.7, ENST00000527343.5, ENST00000584364.5, ENST00000263645.9, ENST00000615999.4 and ENST00000518154.5) mainly regard dendrite regeneration. Therefore, the overall scenario could reveal a dual response by stressed cells: on one hand, the alteration of retinal cell crosstalk and synapses could lead to various forms of cell death (e.g., autophagy). On the other hand, RPE tries to survive, increasing regeneration of capable parts (e.g., dendrites), by synaptic plasticity. Detailed info on pathways enrichment are available in Table S6 and Table S7).

### 3.8. The Most Significant DAS Genes Represented the Main Retinal Dystrophy Candidate Genes

The ToppGene prioritization analysis on known causative retinal dystrophy genes that intersected with most significant DE and DAS genes previously described revealed 19 candidate genes (Bonferroni corrected *p*-value <0.05). Of these, seven showed a strong association with the training genes (Bonferroni corrected *p*-value <0.01). Most of the 19 significant obtained candidate genes, included three of most statistically associated (*PAX6*, *CTSH* and *HNRPA1*) belong to DAS genes, further highlighting the influence of oxidative stress on alternative splicing (Table 1). Details of ToppGene results are available in Table S8.

### 3.9. qRT-PCR Validation

To validate the authenticity and reproducibility of the RNA-Seq results, the 15 selected mRNAs were validated by qRT-PCR analysis, and obtained expression profiles were similar to the results of transcriptome analysis (Table S9). Moreover, the ANOVA statistics, conducted to compare the means among multiple groups, highlighted high significance (*p*-values < 0.05). The linear regression analysis showed a significantly positive correlation of the relationship between gene expression ratios of qRT-PCR and RNA-Seq for all selected time points (Figure S3), confirming our transcriptomic data validity.

## 4. Discussion

Retinal dystrophies like age-related macular degeneration and, particularly, retinitis pigmentosa represent a very heterogeneous group of ocular pathologies characterized by a very complex pattern of environmental and genetic causes. One of the most challenging aspects regards the incomplete knowledge of all causative genes and their involved biochemical and molecular pathways, leading to a huge group of orphan forms [37]. Gene mutations or dysfunctional processes not only in the retina but also in RPE could cause inherited retinal degeneration, age-related macular degeneration and other retinal diseases [38]. Such a feature highlights the relevant role of RPE, a high metabolic demand monolayer of pigmented cells that plays fundamental functions for both rods and cones, such as metabolite transport and photoreceptor excitability, regulation of visual cycle, secretion of growth factors, phagocytosis of photoreceptor outer segments (POSs) and oxidative stress defense. Regarding the latter point, oxidative stress represents one of the major lethal mechanisms responsible for age-related RPE damages [39]. Many studies have demonstrated that accumulation of lipid deposit called lipofuscin generates reactive oxygen species through phototoxicity in RPE cells [40,41,42]. Oxidative stress triggered by photo-oxidation of bis-retinoid A2E, a lipofuscin constituent, is well known to be a progression factor of age-related macular degeneration and also in genetic macular degeneration syndromes such as Stargardt disease [43], but very little is known about A2E involvement in retinitis pigmentosa.

In this study, we treated RPE cells with A2E during a follow-up of two time points (3 h and 6 h) after exposure and compared them to untreated time zero controls. The main purpose of our experiment was the discovery of new pathways potentially involved in retinal dystrophies development, with the further detection of new candidate genes that could be associated or causative of such ocular diseases, emerging from the expression analysis in such altered conditions.

Starting from an initial average value of 16,173 detected genes per sample, about 2432 showed changes in their expression level and 119 were differentially alternative spliced with transiently, late or enduring fluctuations. Selected altered DE and DAS genes were then functionally and statistically analyzed and clustered into final 10 candidate “macro-pathways”, showing a very variable time of exposure-related trends. Considering the average fold-change of each constituting genes and their reciprocal connections, we revealed a more detailed functional network. Such connections could help to depict several causative/associative clusters, underlining a more complex pattern of possible retinal dystrophies etiologies.

From analyses of DE genes related pathways, it emerged that, after an early increase of apoptosis processes, the programmed cell death decreased in both considered time points following A2E exposure, probably due to activation of rescue systems and to a limited percentage of survived cells. An opposite trend was shown by steroid receptor and nucleoside transport activities, which evidenced a huge up-regulation of involved genes after 3 h and 6 h. Such results could reflect late alterations in RPE antioxidant and anti-inflammatory abilities [44], as well as inhibition of photoreceptor outer segment (POS) phagocytosis and impairment of ion currents in retinal cells [45,46]. Furthermore, a very interesting result was the sinusoidal trend involving isoprenoid pathway, related to cholesterol-dependent homeostasis of POS [47] and angiogenesis [48]. After 3 h from treatment, it could be possible that disc bulk membranes increased trying to improve phototransduction by residual photoreceptors, despite the decreased choriocapillaris viability due to the reduced vascular endothelial growth factor (VEGF) [49]. The situation reversed at 6 h observation, when the discs turnover was drastically decreased and the VEGF level increased, which could contribute to the subretinal neovascularization already characterized in wet age macular degeneration [50].

Additionally, the extensive alternative splicing information identified from DAS-related pathway analysis highlighted a much higher degree of complexity of regulation in response to A2E-induced oxidative stress, which has been significantly underestimated by analysis of DE genes only. In particular, speed and extent of the oxidative stress-induced AS suggested that AS, together with the transcriptional response, is a major driver of transcriptome reprogramming for RPE cell death and their attempts to survive. From 3 h and up to 6 h from treatment, an impairment of intracellular traffic related to Rab proteins, already reported in choroideremia [51], was observed along with the alteration of autophagy and accumulation of proteins and damaged organelles. These events are typical of AMD [52] and are also enforced by inactivation of chaperone genes [53,54]. This scenario could reflect a strong reduction of macroautophagy (a catabolic cell survival system) and of a hybrid autophagy–phagocytosis-degradative pathway called LC3-associated phagocytosis (LAP) [55], which plays a critical role in visual pigment regeneration, as well as the complete degradation of phagosomes [56]. The other resulted pathway “big cluster”, instead, highlighted a global up-regulation of DAS-involved genes up to the end of the last considered time point. In particular, we demonstrated the dynamic contribution of AS by changes in multiple different mechanisms of transcription and translation. Photosensitization of A2E stimulates oxidative DNA damage, such as the production of 8-oxo-guanines in telomeres, leading to their possible damage [57]. Thus, the resulted alteration of telomerase RNA localization to Cajal body could accelerate the RPE senescence [58], and this process could be further increased by the reduction of FGFR1 signaling and the consequent POS phagocytosis decrease [59]. Moreover, DNA damage response could increase the activity of miRNAs involved in [60,61] and cell death genes transcription by TP53 [62], determining a possible role in retina degeneration. In the meantime, as already discussed, retinal cells could try to fight against induced stress, and one resistance mechanism could be represented by the improved maturation of the large ribosomal unit (LSU) [63] and by the increased polyribosome activity, showing a fundamental role in translation regulation of many retinal genes [64,65]. Detailed analysis of DE and DAS gene-involved pathways, with their possible impact on retinal dystrophies etiopathogenesis, is reported in Table 2.

## 5. Conclusions

We realized a whole RNA-seq experiment on RPE cells treated with A2E, considering two time points (3 h and 6 h) after the basal one. We found 10 different clusters of pathways involving DE and DAS genes, with many highlighted sub-pathways, which could depict a more detailed scenario determined by induced oxidative stress. Regulation and/or alterations of angiogenesis, extracellular matrix integrity, isoprenoid-mediated reactions, physiological or pathological autophagy, cell death induction and retinal cell rescue represented the most dysregulated pathway, probably involved in retinal degeneration. Assembly of splicing and transcriptional networks from analyzed data will further define the contribution of AS, as an extra level of regulation, and the interplay and coordination of the transcriptional and AS responses. However, it is fundamental to highlight several limitations of our study: RPE-cultured cells were not in contact with photoreceptors’ outer segments and did not perform any phagocytosis. Moreover, the short-term response (at 3 h and 6 h) detected in vitro do not surely reflect what happens in vivo. Finally, even if it was important to underline the importance to realize an in vivo experiment to confirm what observed in RPE cells, our results could represent an important step towards discovery of unclear molecular mechanisms involved in etiopathogenesis of retinal dystrophies.

## Figures and Tables

**Table 1:** ToppGene prioritization analysis results. The ToppGene prioritization analysis on known causative retinal dystrophy genes that intersected with most significant DE and DAS genes obtained during the whole transcriptome analysis revealed 19 candidate genes (Bonferroni corrected *p*-value < 0.05), of which seven had a strong association with training genes (Bonferroni-corrected *p*-value < 0.01). All shown *p*-values are Bonferroni-corrected.

Rank	Gene Symbol	GeneId	GO: Mol. Func. Score	GO: Mol. Func. *p*-Value	GO: Bio. Proc. Score	GO: Bio. Proc. *p*-Value	GO: Cell. Comp. Score	GO: Cell. Comp. *p*-Value	Human Pheno. Score	Human Pheno. *p*-Value	Pathway Score	Pathway *p*-Value	Pubmed Score	Pubmed *p*-Value	Disease Score	Disease *p*-Value	Average Score	Overall *p*-Value
1	PAX6	5080	0.0422	0.0931	1.0	0.0102	0.2814	0.0807	1.0	0.0135	0.0	0.503	1.0	0.001	0.999	7.75 × 10^−10^	0.6176	1 88 × 10^−12^
2	ACTG1	71	0.8143	0.0106	0.996	0.0278	0.9686	0.0127	0.999	0.0135	0.0	0.503	0.330	0.032	0.730	0.006	0.7267	5 15 × 10^−11^
3	TGFBI	7045	0.6329	0.0288	0.982	0.0462	0.4683	0.0534	0.988	0.0331	0.0	0.503	0.204	0.073	0.819	0.003	0.6226	0.002
4	CCN2	1490	0.4318	0.0491	0.999	0.0209	0.4362	0.0544	0.999	0.0253	0.0	0.503	0.204	0.073	0.551	0.011	0.5529	0.003
5	CTSH	1512	0.2796	0.0677	0.999	0.0185	0.9986	0.0036	0.976	0.0358	0.0	0.503	0.0	0.536	0.445	0.016	0.5302	0.003
6	GNAI2	2771	0.9263	0.0029	0.962	0.0573	0.9500	0.0166	0.999	0.0195	0.0	0.503	0.095	0.073	0.0	0.512	0.5951	0.004
7	LTBP2	4053	0.5918	0.0375	0.813	0.1049	0.0350	0.2254	0.999	0.0135	0.0	0.503	0.204	0.073	0.636	0.009	0.5168	0.007
8	HNRNPA1	3178	0.0421	0.0931	0.910	0.0772	0.5481	0.0499	0.999	0.0214	0.0	0.503	0.801	0.006	0.0	0.512	0.4913	0.011
9	GPI	2821	0.0421	0.0931	0.965	0.0563	0.9563	0.0156	0.986	0.0335	0.0	0.503	0.490	0.019	0.0	0.512	0.5249	0.011
10	CD81	975	0.6702	0.0213	0.999	0.0209	0.3057	0.0782	0.999	0.0232	0.0	0.503	0.095	0.073	0.0	0.512	0.4691	0.011
11	FTL	2512	0.2622	0.0766	0.587	0.1403	0.2370	0.0979	0.999	0.0205	0.0	0.503	0.095	0.073	0.331	0.021	0.4151	0.012
12	ITGAV	3685	0.4732	0.0476	0.999	0.0156	0.9885	0.0094	−1.0	0.0	0.0	0.503	0.076	0.073	0.0	0.512	0.4563	0.014
13	CAPZB	832	0.5061	0.0426	0.998	0.0242	0.9934	0.0079	−1.0	0.0	0.0	0.503	0.205	0.073	0.0	0.512	0.4919	0.015
14	LTBP3	4054	0.0421	0.0931	0.982	0.0458	0.0350	0.2254	0.999	0.0178	0.0	0.503	0.0	0.536	0.636	0.009	0.3864	0.018
15	CTSB	1508	0.2996	0.0642	0.915	0.0753	0.3862	0.0600	0.183	0.0553	0.0	0.503	0.343	0.032	0.0	0.512	0.3794	0.026
16	P4HB	5034	0.2996	0.0642	0.851	0.0960	0.2370	0.0979	0.941	0.0400	0.0	0.503	0.351	0.032	0.0	0.512	0.4317	0.031
17	MATN2	4147	0.4747	0.0464	0.987	0.0408	0.3796	0.0606	−1.0	0.0	0.0	0.503	0.204	0.073	0.0	0.512	0.3834	0.042
18	TMEM189-UBE2V1	387522	−1.0	0.0	−1.0	0.0	−1.0	0.0	−1.0	0.0	0.0	0.503	0.059	0.073	0.579	0.011	0.2746	0.050
19	ACADVL	37	0.6272	0.0290	0.903	0.0792	0.2422	0.0857	0.980	0.0348	0.0	0.503	0.0	0.536	0.0	0.512	0.4014	0.053
20	CD151	977	0.3015	0.0639	0.776	0.1113	0.4489	0.0542	0.994	0.0313	0.0	0.503	0.0	0.536	0.0	0.512	0.3755	0.065
21	CAPNS1	826	0.0421	0.0931	0.819	0.1039	0.1428	0.1432	−1.0	0.0	0.0	0.503	0.076	0.073	0.0	0.512	0.2277	0.103
22	CD63	967	0.0	0.5652	0.986	0.0416	0.3390	0.0613	−1.0	0.0	0.0	0.503	0.095	0.073	0.0	0.512	0.2735	0.104
23	RNA5-8SN2	109864281	0.3250	0.0559	−1.0	0.0	0.2466	0.0846	−1.0	0.0	−1.0	0.0	0.0	0.536	−1.0	0.0	0.1905	0.112
24	RPL3	6122	0.3250	0.0559	0.952	0.0609	0.2814	0.0807	−1.0	0.0	0.0	0.503	0.0	0.536	0.0	0.512	0.2637	0.117
25	RPS11	6205	0.3250	0.0559	0.877	0.0861	0.2814	0.0807	−1.0	0.0	0.0	0.503	0.0	0.536	0.0	0.512	0.2571	0.132
26	RPL19	6143	0.3250	0.0559	0.877	0.0861	0.2814	0.0807	−1.0	0.0	0.0	0.503	0.0	0.536	0.0	0.512	0.2571	0.132
27	SLC16A3	9123	0.0	0.5652	0.212	0.2039	0.3057	0.0782	−1.0	0.0	0.0	0.503	0.065	0.073	0.0	0.512	0.1703	0.192
28	UTP14C	9724	0.0	0.5652	0.220	0.2037	0.2814	0.0807	−1.0	0.0	0.0	0.503	0.0	0.536	0.0	0.512	0.1251	0.353
29	TMSB4XP6	7120	−1.0	0.0	−1.0	0.0	−1.0	0.0	−1.0	0.0	−1.0	0.0	0.0	0.536	−1.0	0.0	0.0	1.0

**Table 2:** Detailed analysis of DE and DAS gene-involved pathways, with their possible impact on retinal dystrophies etiopathogenesis. DE and DAS genes were dysregulated during the whole analysis and showed several fluctuations during observed time points, suggesting that changes in gene-level expression and alternative splicing occurred throughout the whole period, either transiently (occurring after 3 h and returning to initial level after 6 h), occurring later (only after 6 h from treatment) or enduring throughout the whole period.

De Gene-Involved Pathways	Expression Changes	Das Gene-Involved Pathways
RNA methyltransferase	3 h and 6 h = DOWN-REGULATED	Endosomal sorting complex required for transport (ESCRT) and RAB geranylgeranylation
TRAF6 mediated NF-kB activation and activation of IKK by MEKK1		Phagopore assembly site membrane; C-terminal protein lipidation; protein localization to microtubule cytoskeleton; regulation of TNFR1 signaling; TNF signaling
Condensed chromosome outer kinetochore and kinesin complex		Methylation; activation of chaperone genes; nucleotide-sugar biosynthetic process
Transport of nucleoside and free purine and pyrimidine; histone pre-mRNA DCP binding; formation of AT-AC complex; respiratory electron transport	3 h and 6 h = UP-REGULATED	Negative regulation of FGFR1 signaling
		Regulation of telomerase RNA localization to Cajal body; maturation of LSU-rRNA; miRNAs involved in DNA damage response
		Mithocondrial intermembrane space and TP53 regulates transcription of cell death genes
		Polysomal ribosome
Mevalonate pathway and cholesterol biosynthesis	3 h = UP-REGULATED 6 h = DOWN-REGULATED	Cholesterol biosynthesis
CBL binds and ubiquinates Sprouty and MAP kinase phosphatase activity	3 h = DOWN-REGULATED 6 h = UP-REGULATED	/
PTK2/SRC-1 phosphorylates BCAR1		

## Supplementary Materials

The following are available online at https://www.mdpi.com/2076-3921/9/4/307/s1, Figure S1: Time-series trend analysis, Figure S2: Gene expression data statistics, Figure S3: qRT-PCR validation of ten most differentially expressed genes, Table S1: Gene expressed in RPE cells transcriptome analysis, Table S2: Gene transcripts expressed in RPE cells transcriptome analysis, Table S3: DE genes profile of RPE Cells, Table S4: DAS genes profile of RPE Cells, Table S5: DTU transcripts profile of RPE Cells, Table S6: Significant DE vs DAS gene enrichment by DAVID, Table S7: Significant DE vs DAS gene enrichment by ClueGO, Table S8: ToppGene prioritization detailed results, Table S9: Expression profiles of 10 among most DE genes.
