# Supplementary material for: Effects of A2E-Induced Oxidative Stress on Retinal Epithelial Cells: New Insights on Differential Gene Response and Retinal Dystrophies
Source: Antioxidants (Basel). 2020 Apr 10;9(4):307. doi: 10.3390/antiox9040307 (PMC7222197; doi:10.3390/antiox9040307)
Supplement: Supplementary file 1 [file antioxidants-09-00307-s001.zip › Figures S1-S3_Revised.pdf]

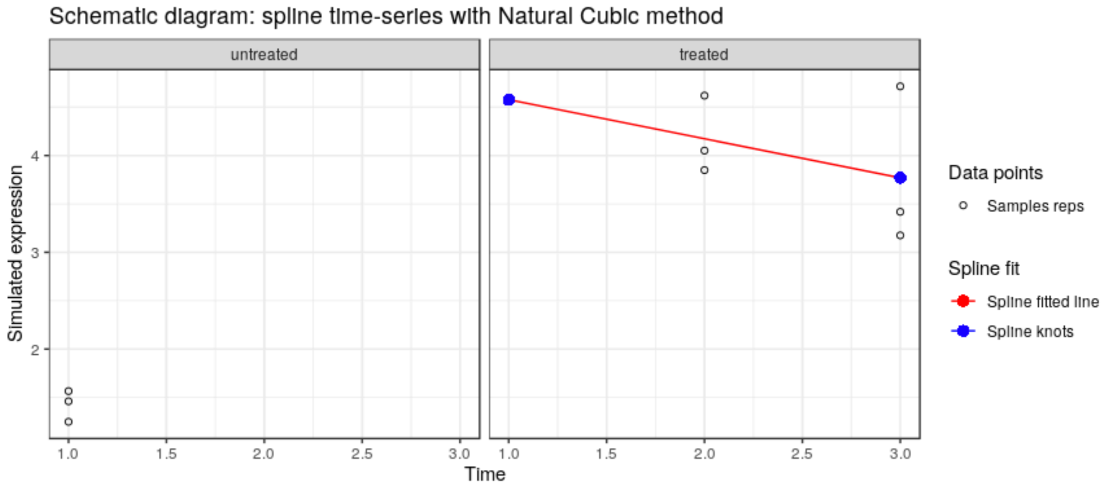

Figure S1. Time-series trend analysis. Considered time-points: (Basal, 3h and 6h) for analyzed groups (basal untreated and treated) were used for 3D trend analysis. Natural Cubic Spline method with degree of freedom was used to generate time-series trend. Significant 3D time-series trend genes and transcripts were determined by BH adjusted p-value < 0.01. At alternative splicing level, the p-value of a DAS gene was summarised from the p-values of transcript level DTU analysis with F-test method. The overall DAS gene/DTU transcript p-values across time groups in each of the contrast groups were summarised by Simes method.

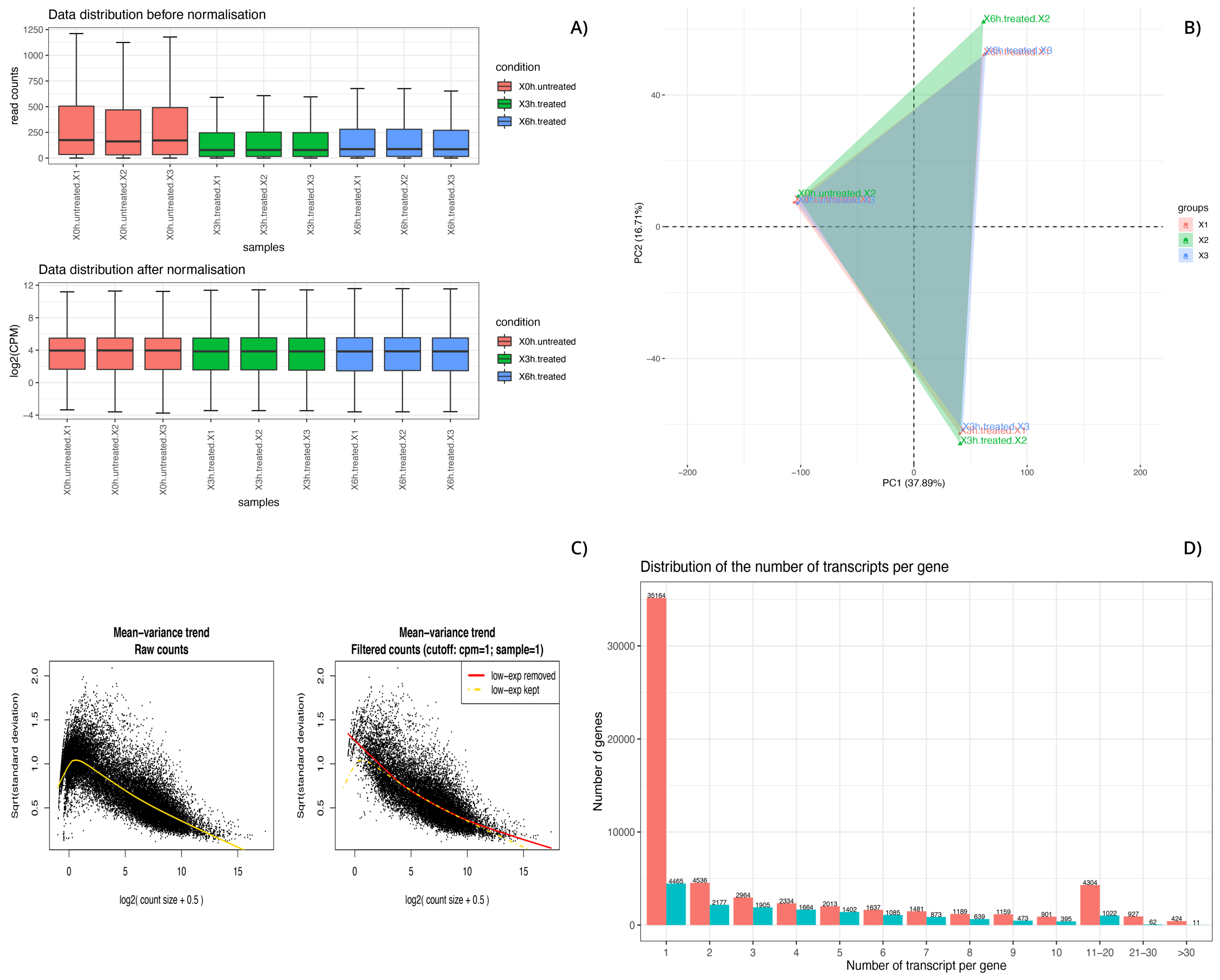

Figure S2. Gene expression data statistics. A) Gene level read counts and normalised log_2-CPM distribution across samples; B) PCA plot based on normalised log_2-CPM of all Biological Replicates; C) Gene level mean-variance trend plot; D) Distribution of the number of transcripts per gene. Low expressed transcripts and genes are filtered.

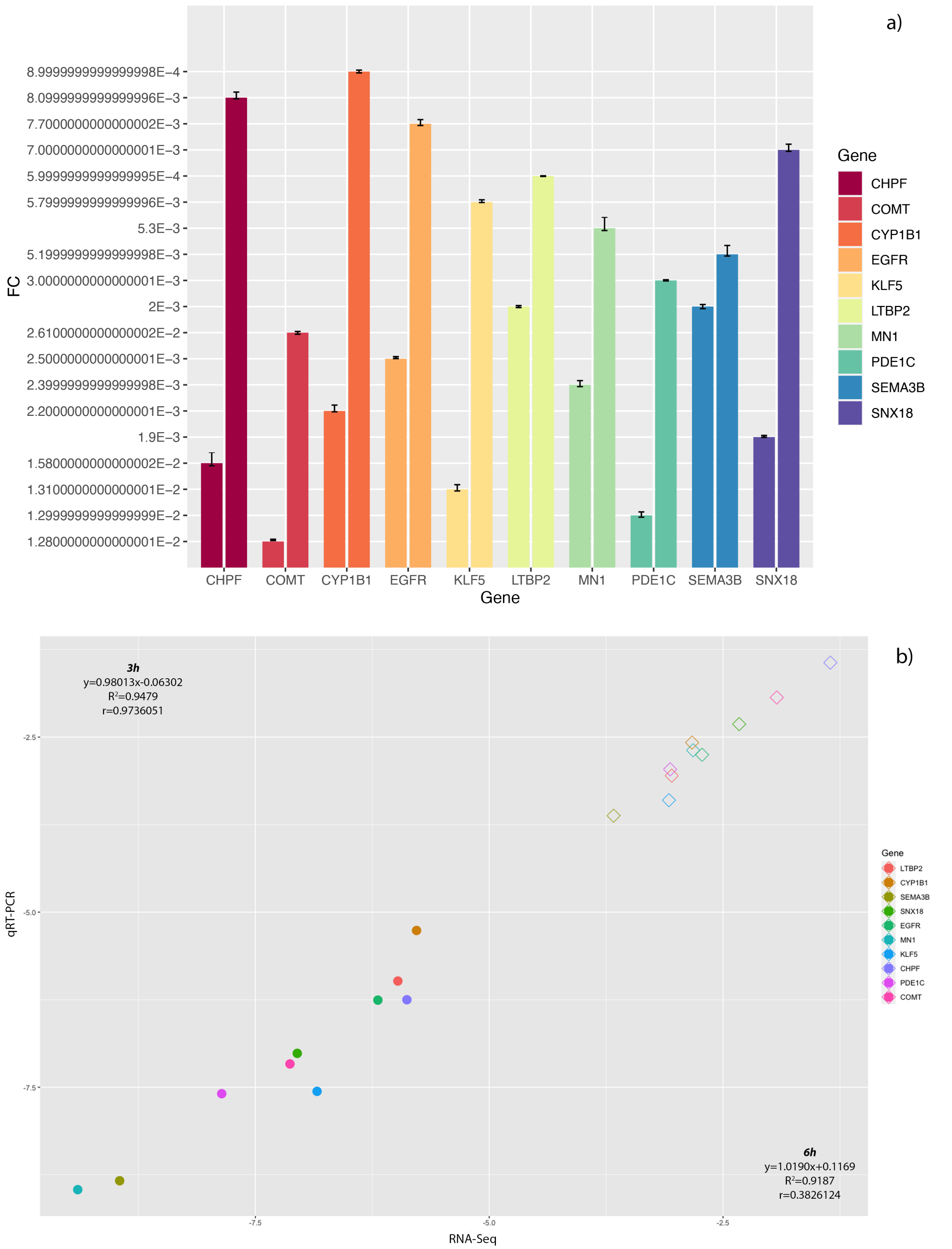

Figure S3. qRT-PCR validation of ten most differentially expressed genes. Histograms reporting the relative expression values, in form of fold change (FC) computed by $2^{-\Delta\Delta Ct}$ method, of ten chosen differentially expressed genes obtained by qRT-PCR experiments, comparing A2E-treated samples (3h and 6h) with basal

time point, and considering six replicates for each condition (mean values were reported). Obtained results were statistically significant (ANOVA Bonferroni-corrected p-values < 0.05). b) Correlation plot between RNA-Seq log2FC and qRT-PCR log2FC data, as mean of all considered replicates, confirmed the RNA-Seq result validity. Empty Diamond = RNA-Seq log2FC value for that gene at 3h (3h vs basal time). Circle = qRT-PCR log2FC value for that gene at 6h (6h vs basal time).
